# Crossreactive Autoantibodies Directed against Cutaneous and Joint Antigens Are Present in Psoriatic Arthritis

**DOI:** 10.1371/journal.pone.0115424

**Published:** 2014-12-16

**Authors:** Marzia Dolcino, Claudio Lunardi, Andrea Ottria, Elisa Tinazzi, Giuseppe Patuzzo, Antonio Puccetti

**Affiliations:** 1 Institute Giannina Gaslini, Genova, Italy; 2 Department of Medicine, University of Verona, Verona, Italy; 3 University of Genova, Genova, Italy; Institute of Immunology, Rikshospitalet, Norway

## Abstract

**Background:**

Psoriatic arthritis (PsA) is a chronic inflammatory disease of unknown origin, characterized by erosions and new bone formation. Diagnosis of PsA is mainly clinical and there are no biomarkers available. Moreover in PsA autoantibodies have not been described so far. Indeed an autoimmune origin has been suggested but never proven. Aim of the study was to investigate the possible presence of autoantibodies typically associated with PsA.

**Methods:**

We used pooled IgG immunoglobulins derived from 30 patients with PsA to screen a random peptide library in order to identify disease relevant autoantigen peptides.

**Results:**

Among the selected peptides, one was recognised by nearly all the patients’ sera. The identified peptide (PsA peptide: TNRRGRGSPGAL) shows sequence similarities with skin autoantigens, such as fibrillin 3, a constituent of actin microfibrils, desmocollin 3, a constituent of the desmosomes and keratin 78, a component of epithelial cytoskeleton. Interestingly the PsA peptide shares homology with the nebulin-related anchoring protein (N-RAP), a protein localized in the enthesis (point of insertion of a tendon or ligament to the bone), which represents the first affected site during early PsA. Antibodies affinity purified against the PsA peptide recognize fibrillin, desmocollin, keratin and N-RAP. Moreover antibodies directed against the PsA peptide are detectable in 85% of PsA patients. Such antibodies are not present in healthy donors and are present in 13/100 patients with seroposive rheumatoid arthritis (RA). In seronegative RA these antibodies are detectable only in 3/100 patients.

**Conclusions:**

Our results indicate that PsA is characterized by the presence of serum autoantibodies crossreacting with an epitope shared by skin and joint antigens.

## Introduction

Psoriatic arthritis (PsA) is characterised by inflammation of entheses and synovium, eventually leading to joint erosions and new bone formation [1]. It affects approximately 10% to 30% of patients with psoriasis, and has an estimated prevalence of approximately 1% [2]. Despite considerable heterogeneity in the presentation of arthropathy and the extent of skin disease, PsA is considered a distinct disease entity with a strong heritable component [3] and several genetic loci have been associated with the disease [4], [5]. PsA shows different clinical phenotypes: oligoarticular or polyarticular asymmetrical peripheral joint inflammation or axial involvement. Various criteria have been proposed to aid the diagnosis and classification of PsA. Although none of them are accepted unequivocally, the classification criteria described by Moll and Wright [6] and more recently the classification criteria for PsA (CASPAR) [7] are the most frequently used. There is no definitive diagnostic test for psoriatic arthritis. The diagnosis is made mostly on a clinical basis and by a process of exclusion of other types of seronegative arthritis. Medical history, physical examination, blood tests, and radiography of the joints may be used for diagnostic purposes. Conventional radiographs have traditionally been used to detect and estimate the extent of joint damage. However newer imaging techniques such as magnetic resonance imaging (MRI) provide the ability to detect joint damage earlier and to measure the extent of joint involvement more accurately than conventional radiographs. MRI allows visualization of soft tissues and articular lesions, thus providing a unique picture of the disease process that cannot be gained using classical imaging modalities. This technique is able to reveal the presence of enthesitis even in apparently unaffected joints and in the absence of clinical symptoms. Enthesitis is the hallmark of PsA and is considered the primary event in the pathogenesis of the disease [8]. At present there are no specific markers that can help in the diagnostic work up and that can accurately predict disease progression and therapeutic response. Moreover, a biomarker able to distinguish between different clinical phenotypes of PsA, or that could be used as a predictive marker for future PsA development in patients with psoriasis, is still lacking.

Therefore, biomarkers useful both in the diagnosis of the disease and in the prediction of response to treatment are needed in order to help clinicians to improve patient management and outcomes. Although many efforts have been made to identify PsA biomarkers none of them has yet been translated into routine clinical practice and so far only acute phase reactants may be used in monitoring the disease activity [9], [10]. Moreover antibodies specific for PsA have not been identified yet. Therefore, the identification of a serologic marker typical of PsA remains a major goal in clinical research. PsA has always been considered of autoimmune origin, driven by autoreactive T cells directed against autoantigens present in the skin and in the joints. This view has been recently questioned by McGonagle et al. who have proposed that PsA may be considered an autoinflammatory rather than an autoimmune disease [11]. One of the reasons adduced is that the autoimmune model would fail to explain diffuse enthesitis, and that the proposed autoantigens common to skin and joint have not been identified so far. In order to address this issue and to identify a potential serologic marker of the disease to be validated as a diagnostic tool, we used a molecular biology approach that has already been successfully applied to other autoimmune disease [12]–[15].

## Methods

### Patients

A written informed consent was obtained from all the participants to the study. The study was approved by local Ethical Committee of the Azienda Ospedaliera Universitaria of Verona, Verona, Italy. All clinical investigation have been conducted according to the principles expressed in the Helsinki declaration. We studied a cohort of 100 patients (64 males and 36 females, mean age: 57±14 years) affected by PsA, attending the Unit of Autoimmune Diseases at the University Hospital of Verona, Italy. All patients fulfilled the CASPAR criteria for the diagnosis of PsA: inflammatory musculoskeletal involvement (inflammatory arthritis, enthesitis or lumbar pain) combined with at least 3 points: 1) evidence of current psoriasis, personal history of psoriasis, family history of psoriasis in unaffected patiens; 2) affected nails (onycholysis, pitting); 3) dactylitis; 4) negative rheumatoid factor; 5) radiographic evidence of new juxta-articular bone formation (excluding osteophytes) [16]. We considered the age at diagnosis (43±7 years) and calculated the interval time from the PsA onset to diagnosis (3±1,5 years). All the patients underwent clinical examination and laboratory evaluation comprehensive of inflammatory markers, such as CRP and erythrocytes sedimentation rate (ESR); rheumatoid factor (RF) and anti-CCP antibody detected by ELISA test; antinuclear antibody detected by indirect immunofluorescence on HEp-2 cells; and genetic screening for the association with haplotype HLA-B27. All patients underwent the following instrumental investigations: ultrasonography with Power Doppler to investigate subclinical enthesopaty and synovitis in asymptomatic patients; conventional radiography, magnetic resonance imaging (MRI) and scintigraphy. The clinical features of the patients are reported in the Table 1. Fifty age- and sex-matched healthy subjects served as control group (38 males and 12 females, age 54±10). Moreover 200 patients with rheumatoid arthritis (RA), 30 patients with systemic lupus erythematosus (SLE: 27 females and 3 males, age 45±15), 30 patients affected by Sjogren’s syndrome (SS: 28 females and 2 males, age 55±14), 30 patients with systemic sclerosis (SSc: 24 females and 6 males, age 52±11), 30 patients with ankylosis spondylitis (24 males and 6 females, age 50±16) and 30 patients affected by psoriasis without arthropathy (21 males and 9 females, age 53±12) were used as controls. RA patients met the American College of Rheumatology classification criteria for RA [17]. All RA patients had active disease, and blood was collected before starting therapy with anti-TNF agents. RA patients were divided into two groups: 100 seropositive patients (62 females and 38 males, age 56±15) and 100 seronegative patients (66 females and 34 males, age 58±17) on the basis of rheumatoid factor (RF) and anti-CCP antibodies detected by ELISA test. All the other patients used as controls met the classification and diagnostic criteria for the disease [18]–[21]. All the enrolled patients and healthy subjects were of Caucasian origin from Northern Italy.

**Table 1:** Clinical features of PsA patients enrolled in the study.

Patients 100		
Sex	male	64
	female	36
Age at diagnosis (years)		43±7
Age at enrollment (years)		57±14
Time from onset of symptoms to diagnosis (years)		3±1,5
Involvement	axial	41
	peripheral	59
Enthesitis		48
Dactylitis		39
Psoriasis		79
Association with HLA-B27		30

### Peptide Library

A random dodecamer peptide library that shows peptides on the surface of *Escherichia coli* was purchased from Invitrogen and screened with pooled immunoglobulins purified from the serum samples from the 30 patients with PsA, according to the manufacturer’s instructions (FliTrx Panning Kit, Invitrogen, Carlsbad, California, United States). After five rounds of biopanning experiments, the enriched library was grown, and single colonies were induced with tryptophan to express the fusion peptides. Bacteria were lysed in sample buffer and tested by means of Western blotting with the pooled immunoglobulin fraction from the patients with PsA in order to check for positive clones. DNA was extracted from positive clones and sequenced. A set of 12 of the 27 peptides obtained from the last biopanning round was synthetized and used in a dissociation-enhanced lanthanide fluorescence immunoassay (DELFIA) to test serum specimens from individual patients.

### Peptide Synthesis

All the synthetic peptides, including the PsA peptide (TNRRGRGSPGAL), the N-RAP [22], [23] peptide (EQQRGKGSFPAM) and the irrelevant control peptide (VTLPKDSDVELP), were manually synthesized by means of the standard method of solid-phase peptide synthesis; this method uses the 9-fluorenylmethoxycarbonyl strategy with minor modifications [24].

### Assessment of Antibody Binding

The DELFIA is a time-resolved fluorescence method that can be used to study antibody binding to solid-phase proteins or peptides. The peptides were used at a concentration of 20 µg per milliliter in phosphate-buffered saline to coat DELFIA plates (PerkinElmer, Norwalk, Connecticut, United States). Plates were then blocked for 1 hour with a blocking reagent (PerkinElmer). Serum samples were diluted in a 1∶50 solution in phosphate-buffered saline plus 1% bovine serum albumin (Sigma, St. Louis, Missouri, United States) and incubated on the plates overnight at 4 to 8°C. Plates were then washed 10 times with washing buffer (PerkinElmer). Bound antibodies were detected with europium-labeled antihuman IgG antiserum (1∶500 in diluting buffer, PerkinElmer). Plates were read on a Victor3 instrument (PerkinElmer), and the data were analyzed with software supplied with the DELFIA instrument. Absorbance values higher than the mean (+3 SD) of the control group were considered to be positive. For DELFIA with recombinant proteins all the proteins were used at a concentration of 10 microgram/ml. Fibrillin 3 and keratin were purchased from Antibodies Online, desmocollin III was purchased by Abcam (Cambridge, United Kingdom). Recombinant IL17B was purchased from Abcam and used at 20 microgram/ml in the coating solution. For competitive assays the antibody concentration that gave 50% of the maximal binding to the solid phase antigen was preincubated for 1 hr at 37°C in the presence of different concentrations of inhibitors and then transferred to an already coated DELFIA plate. The remainder of the assay was then performed as above.

### Affinity Purification of Anti-Peptide Antibodies

Synthetic peptides (5 mg peptide per gram of dried Sepharose powder) were coupled to Sepharose 4B (Pharmacia, Uppsala, Sweden) according to the manufacturer’s instructions. Serum Igs diluted in PBS were applied to the columns. The columns were washed with PBS. Bound Ig were eluted with 0.1 M glycine (pH 2.5) and dialyzed against PBS. The purity of the preparations was assessed by SDS-PAGE.

### Western Blotting

To detect binding of purified anti-PsA peptide antibodies to TLR2, HEK cell lysates were prepared with the use of a commercially available kit (Nuclear Extract Kit, Active Motif, Carlsbad, CA United States). Cell lysates were immunoprecipitated with rabbit antibody directed against human TLR2 (Abcam) cross-linked to Sepharose. Eluted proteins were resolved by means of sodium dodecyl sulfate–polyacrylamide-gel electrophoresis (SDS-PAGE) and transferred onto a nitrocellulose membrane (Amersham Biosciences, Piscataway, New Jersey, United States). Blots were probed with primary antibodies followed by peroxidase-linked anti-human immunoglobulin antibodies (Sigma). In another set of experiments HEK cell lysates were were resolved by SDS-PAGE and transferred onto nitrocellulose paper. Blots were probed with a commercially available monoclonal antibody directed against TLR2 (Abcam) followed by peroxidase-labeled anti-mouse immunoglobulin antibodies.

### Statistical Analysis

All the calculations were performed with SPSS 21.0 statistical package (SPSS Inc., Chicago, IL, USA) using the Chi-square statistical test. A value of *P*<0.05 was cosidered statistically significant.

## Results

### Identification of an autoantigen peptide recognized by serum immunoglobulins of patients with PsA

We screened a dodecamer random peptide library with pooled immunoglobulins derived from 30 patients with recent onset PsA. A set of 12 peptides, out of the 25 peptides obtained from the last biopanning round, was synthetized and used to test the individual patients’sera in a DELFIA assay employing the solid phase peptide. By this approach, we identified a peptide called PsA peptide (TNRRGRGSPGAL) that was recognized by serum IgG of 86% (26/30) individual patients, by DELFIA; such reactivity was not detected in the sera of 50 age- and sex-matched healthy controls. In an additional validation panel of 70 PsA patients, whose sera were not used for the library screening, anti–PsA peptide antibodies have been detected in 59/70 patients’sera. Moreover only 1/30 patients with psoriasis had IgG antibodies directed against the PsA peptide. These data indicate that the PsA peptide contains an epitope recognized by the vast majority of sera from patients with PsA.

### Target autoantigens in PsA

As PsA is characterized by articular and cutaneous damage, we next compared the PsA peptide sequence with human proteins in a protein data bank (Swiss-Prot database) using the BLASTP via the NCBI BLAST network service to identify potential autoantigens (human proteins) sharing homology with the PsA peptide. Two criteria were used in selecting the homologous autoantigen peptides: the first is the extent of homology obtained by measuring the length of the homologous stretch and the number of matching amino acids (both identities and conservative substitutions were included); the second one depends on the characteristics of the autoantigen targets. We privileged those proteins that are present in the two main affected sites in PsA, ie skin and joints. To this aim the entire group of homologous peptides was further “filtered” through the NCBI DELTA BLASTP by narrowing the web search to skin and joint proteins. Using this approach we found that the peptide shares homology with several autoantigen proteins (Table 2): a) fibrillin 3, which is localized in actin microfibrils of skin [25]; b) desmocollin 3, a calcium-dependent glycoprotein of the desmocollin subfamily localized in epithelial cell junctions where it constitutes, together with desmogleins, the adhesive proteins of the desmosome [26] and c) keratin 78, a member of the type II keratin family with an intermediate filament domain (Table 2). The same peptide also shares homology with a protein called nebulin related anchoring protein (N-RAP) [22], [23], a protein highly expressed at the sites of insertion of a tendon or ligament into the bone (enthesis). N-RAP has an anchoring function linking the terminal actin filaments to protein complexes beneath the plasma membrane and functions in transmitting tension from the inside cytoskeleton to the extracellular matrix.

**Table 2:** Peptides used in the study and sequence homologies.

PsA peptide	T	N	R	R	G	R	G	S	P	G	A	L
				:		:	:	:	:	:		:
Fibrillin-3 (46–54)				R	R	R	G	S	P	G	I	L
PsA peptide	T	N	R	R	G	R	G	S	P	G	A	L
		:			:	:	:	:	:			
Desmocollin-3 (853–860)		N	Y	E	G	R	G	S	P			
PsA peptide	T	N	R	R	G	R	G	S	P	G	A	L
		:	:		:		:					
Desmocollin-3 (500–505)		N	R	N	G	N	G					
PsA peptide	T	N	R	R	G	R	G	S	P	G	A	L
					:	*	:	:	:	:	*	
Keratin-78 (460–466)					G	K	G	S	P	G	S	
PsA peptide	T	N	R	R	G	R	G	S	P	G	A	L
			*	:	:	*	:	:			:	*
Nebulin related anchoring protein N-RAP (235–244)			Q	R	G	K	G	S	F	P	A	M
PsA peptide	T	N	R	R	G	R	G	S	P	G	A	L
				:	:	*	:	:			:	*
Nebulin related anchoring protein N-RAP (306–314)				R	G	K	G	S	F	P	A	M
PsA peptide	T	N	R	R	G	R	G	S	P	G	A	L
	*		:	*	:	*	:		:	:		:
IL17B (27–38)	S	K	R	K	G	Q	G	R	P	G	P	L
PsA peptide	T	N	R	R	G	R	G	S	P	G	A	L
						*	:	:		:	*	:
TLR2 (37–43)						K	G	S	S	G	S	L

We purified antibodies directed against the PsA peptide from individual sera of 5 PsA patients by affinity chromatography using peptide-Sepharose columns in order to establish whether the purified anti-PsA peptide antibodies were able to recognize the native autoantigen proteins (Fibrillin, desmocollin, and Keratin). For this purpose we used the commercially available recombinant versions of desmocollin 3 (obtained from Abcam), fibrillin 3 and keratin 78 (both purchased from Antibodies Online) as solid phase antigens adsorbed onto DELFIA plates. Since a recombinant version of the N-RAP protein was not available, in this particular case we tested the affinity purified anti-PsA peptide antibodies in a DELFIA assay using the N-RAP peptide (EQQRGKGSFPAM) adsorbed on the plates.

**Figure 1:**
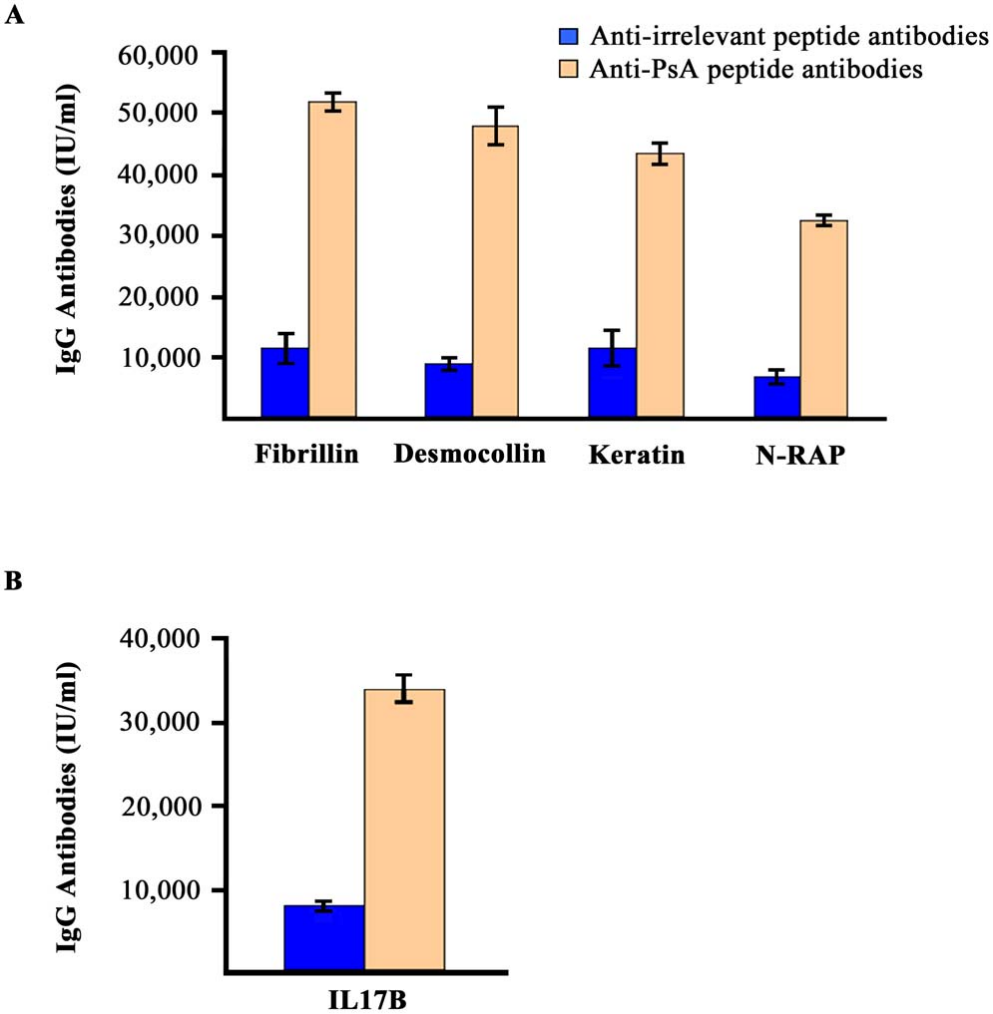
Binding of purified anti-PsA peptide antibodies to autoantigens. **A.** Binding of affinity purified anti-PsA peptide antibodies to solid phase fibrillin 3, desmocollin 3, keratin 78 and N-RAP peptide in DELFIA assay. Fibrillin, desmocollin and keratin are commercially available recombinant molecules. Binding of antibodies to N-RAP was assessed using solid phase N-RAP peptide. The pink bars represent the means of the 5 individual affinity purified anti-PsA peptide antibodies (10 microgram/ml) purified from 5 different PsA patients, the blue bars represent the means of the 5 control antibody populations affinity purified directed against irrelevant peptide from the sera of the same 5 PsA patients. Error bars represent the standard deviations obtained, each test was perfomed in triplicate and repeated three independent times. Y axis absorbance values. **B.** Binding of affinity purified anti-PsA peptide antibodies to solid phase IL17B (see above).

**Figure 2:**
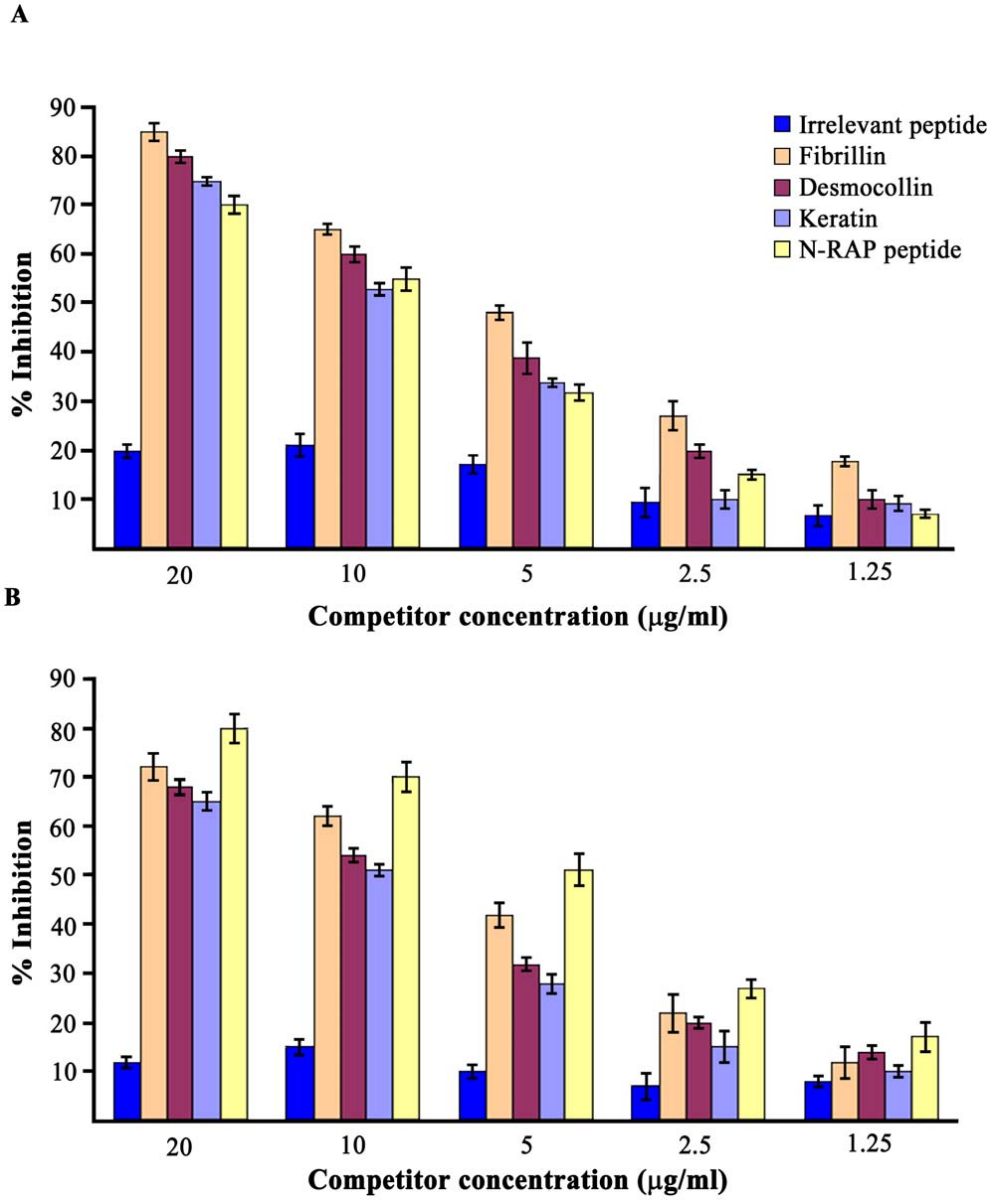
Anti-peptide antibodies crossreact with cutaneous and joint autoantigen. **A.** The binding of affinity purified anti-PsA peptide antibodies to solid phase fibrillin is competed by liquid phase irrelevant control peptide (blue bar), fibrillin (orange bar), desmocollin (magenta bar), keratin (light blue bar) and N-RAP peptide (yellow bar). Anti-peptide antibodies were affinity purified from 5 patients affected by PsA, Each bar represents the mean +/− SD of % binding inhibition of affinity purified anti-peptide antibody preparation from the five patients with PsA. Each experiment was performed in triplicates and repeated 5 different times. **B.** The binding of affinity purified anti-N-RAP peptide antibodies to solid phase N-RAP peptide is displaced by liquid phase irrelevant control peptide (blue bar), fibrillin (orange bar), desmocollin (magenta bar), keratin (light blue bar) and N-RAP peptide (yellow bar). X axis indicates the concentration (µg/ml) of competitor. Y axis: percent of inhibition.

**Figure 3:**
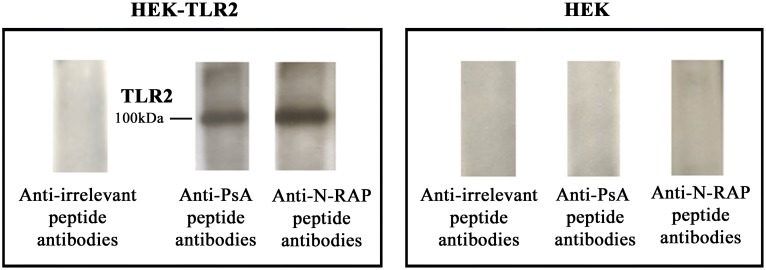
Affinity purified anti-PsA antibodies bind TLR2. Western blot analysis of the binding of affinity purified anti-irrelevant peptide antibodies, affinity purified anti-PsA peptide antibodies or antibodies affinity purified against the N-RAP peptide to TLR2. Cell lysates were immunoprecipitated with a commercially available rabbit antibody against human TLR2 (Abcam) cross-linked to Sepharose. Eluted proteins were resolved with SDS-PAGE and transferred onto a nitrocellulose membrane. Blots were probed with anti-peptide antibodies followed by peroxidase-linked anti-human immunoglobulin antibodies. Left panel shows the experiment performed on HEK cells transfected with *TLR2,* the right panel contains untrasfected HEK2 cells. The blot containing HEK cells probed with a monoclonal antibody directd against TLR2 is included in the Supporting information section.

The affinity purified antibodies bound recombinant fibrillin, desmocollin, keratin and N-RAP peptide in DELFIA (Fig. 1A). We then performed crossinhibition studies and found that the binding of purified anti-PsA peptide antibodies to the solid phase fibrillin was competed by liquid phase fibrillin, desmocollin, keratin and N-RAP peptide (Fig. 2A). We next purified antibodies from PsA patients’ sera using a N-RAP peptide-sepharose column and performed the same experiment above described. The binding of anti-N-RAP peptide antibodies to solid phase N-RAP peptide was competed by N-RAP peptide, fibrillin, desmocollin, and keratin (Fig. 2B).These data indicate that PsA serum contains antibodies able to react with several autoantigens expressed in the skin and in the joint (enthesis). Table 2 lists other two interesting sequence similarities between the identified PsA peptide and two crucial molecules of the immune response. First of all the PsA peptide shares homology with human interleukin 17B (IL17B), an important cytokine of the IL17 family, sharing sequence similarity with IL17A. This cytokine was reported to stimulate the release of TNF alpha (TNF) and IL1 beta (IL1B) and is produced at high concentration by neutrophils infiltrating the synovial membranes of RA patients [27]. Binding of purified anti-PsA peptide antibodies to IL17B was performed by DELFIA using solid phase recombinant human IL17B (Abnova) (Fig. 1B). Finally the PsA peptide shares homology with TLR2, an innate immune receptor, involved in the recognition of a vast array of microbial products deriving from Gram-positive and negative bacteria, mycoplasma and yeasts. Antibodies affinity purified against the PsA peptide recognize TLR2 in western blot using the HEK 293 cell line expressing TLR2 (Fig. 3). As positive control, HEK cell line transfected with *TLR2* were probed with commercially available mouse monoclonal antibodies directed against TLR2 (S1 Figure).

### Anti-PsA peptide antibodies in the sera of patients affected by rheumatic diseases

**Figure 4:**
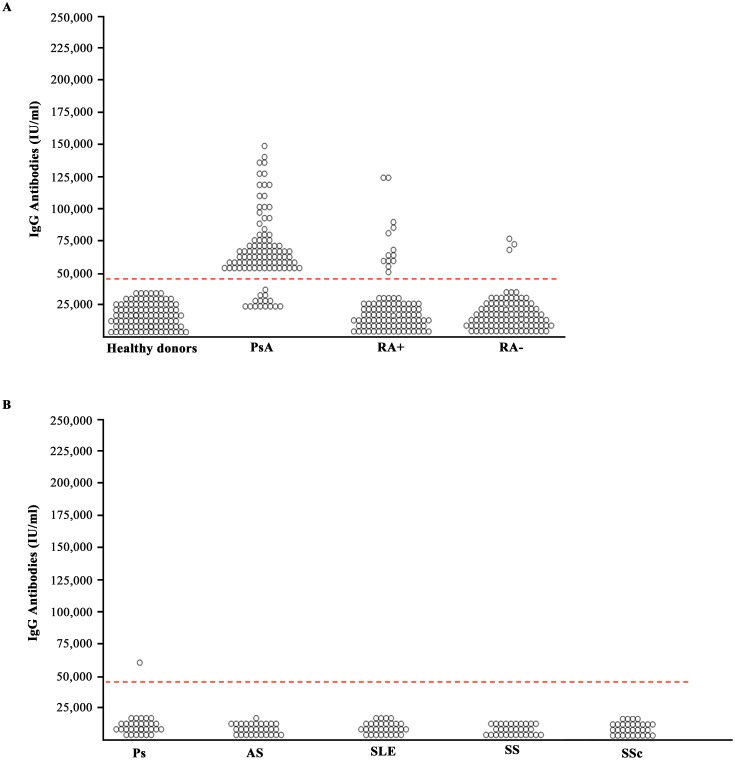
Sera from patients with PsA recognize PsA peptide. Binding to PsA peptide by serum samples of 100 healthy donors, of 100 patients with PsA, of 100 patients with seropositive Rheumatoid Arthritis (RA+), and of 100 patients with seronegative Rheumatoid Arthritis (RA–), of 30 patients with Psoriasis (Ps), of 30 patients with Ankylosing Spondilitis (AS), 30 patients with systemic lupus erythematosus (SLE), 30 patients with Sjogren syndrome and 30 patients with Systemic sclerosis (SSc). The *p* values obtained comparing the percent of antibody positivity were the following: 1) PsA vs healthy donors: P<0.001, 2) PsA vs RA+: P = 0.049; 3), PsA vs RA–: P = 0.005; 4) PsA vs Ps: P = 0.007; 5) PsA vs AS, SLE, SS, SSc: P<0.001. The cut-off value was equal to 46,000 IU/microliter.

Anti-PsA peptide antibodies were detectable in 85/100 (85%) patients with PsA, in 13/100 (13%) patients with seropositive RA and in only 3/100 (3%) patients with seronegative RA (Fig. 4A). The results indicate that anti-PSA peptide antibodies are positive in the vast majority of PsA sera. The assay does not completely discriminate PsA sera from RA sera, however in seronegative RA anti-PsA peptide antibodies are present in 3% of the subjects whereas anti-PsA peptide antibodies can be detected in 13% of patients with seropositive RA. Needless to say that in this case the detection of anti-citrullinated peptides antibodies or the presence of Rhematoid factor, that are negative in PsA, will allow to discriminate the two diseases. Anti-PsA antibodies were present in 1/30 (3.3%) patients with psoriasis (Ps) and were not detected in the sera of patients with systemic sclerosis (0/30 pts), Sjogren syndrome (0/30 pts), systemic lupus erythematosus (0/30 pts) and ankylosing spondylitis (0/30 pts) (Fig. 4B). Since the PsA peptide shares homology with a protein called nebulin related anchoring protein (N-RAP), a protein that is highly expressed in the enthesis, we decided to check whether we can detect antibodies directed against this enthesis-related protein, N-RAP in the sera of patients with PsA.

**Figure 5:**
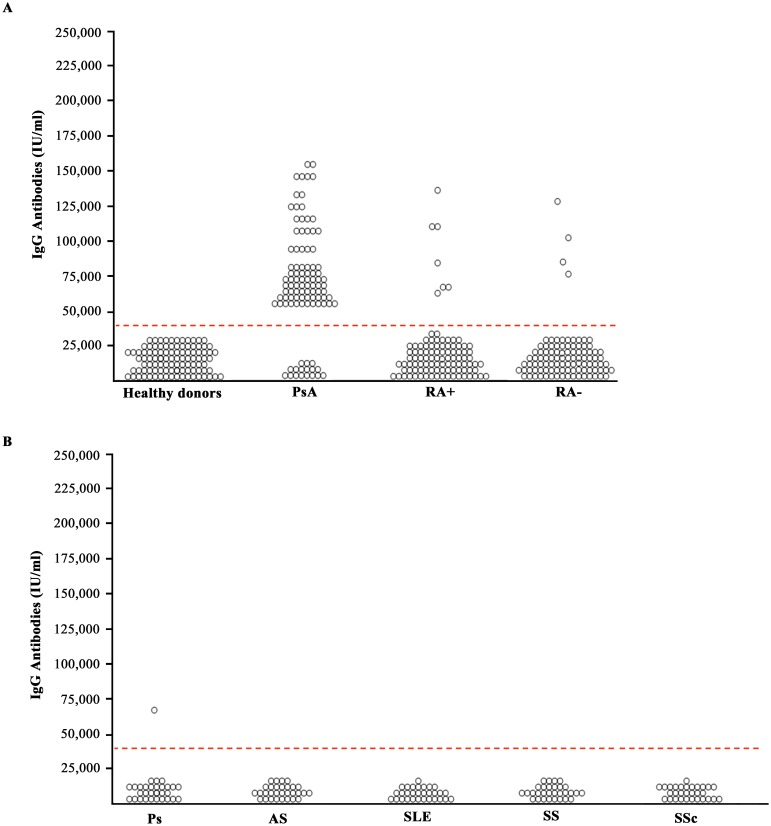
Sera from patients with PsA recognize N-RAP peptide. Binding to N-RAP peptide by serum samples of 100 healthy donors, of 100 patients with PsA, of 100 patients with seropositive Rheumatoid Arthritis (RA+), and of 100 patients with seronegative Rheumatoid Arthritis (RA–), of 30 patients with Psoriasis (Ps), of 30 patients with Ankylosing Spondilitis (AS), 30 patients with systemic lupus erythematosus (SLE), 30 patients with Sjogren syndrome and 30 patients with Systemic sclerosis (SSc). The *p* values obtained comparing the percent of antibody positivity were the following: 1) PsA vs healthy donors: P<0.001, 2) PsA vs RA+: P = 0.004; 3) PsA vs RA–: P = 0.0038; 4) PsA vs Ps: P = 0.001; 5) PsA vs AS, SLE, SS, SSc: P<0.001. The cut-off value was equal to 42,000 IU/microliter.

For this purpose we synthetized a peptide corresponding to the N-RAP sequence (EQQRGKGSFPAM) sharing homology with the PsA peptide and used it to test sera of patients and control subjects. Fig. 5 shows the results obtained. Anti-NRAP peptide antibodies were detected in 83/100 (83%) of the patients with PsA, in 7/100 (7%) patients with seropositive RA and in 4/100 (4%) patients with seronegative RA (Fig. 5A). No reactivity against the N-RAP peptide was detected in systemic sclerosis, Sjogren syndrome, systemic lupus erythemathosus and ankylosing spondylitis, while one out of thirthy sera of patients affected by psoriasis (Ps) bound the N-RAP peptide (Fig. 5B). In conclusion we report here for the first time the presence of autoantibodies directed against autoantigens localized within the skin and the entheses in the sera of patients with PsA. Such autoantigens share common epitopes against which crossreactive antibodies are produced and this may be the missing link that explains the onset of enthesitis in patients with skin psoriasis.

## Discussion

We describe here the identification of a serologic marker that is present in most patients with PsA. In clinical practice, the diagnosis of PsA is based on clinical history and so far there are no biomarkers available to help in the diagnosis of the disease. Moreover, despite extensive investigation, no autoantigens common to skin and enthesis have been identified so far, leading to the hypothesis that PsA may be autoinflammatory in origin. In this regard the identification of epitopes shared by skin and joint proteins and of a novel cross-reactive antibody recognizing these antigens, associated with PsA, sustains the original hypothesis that PsA is of autoimmune origin.

The anti–PsA peptide antibodies we describe here, were undetectable in healthy controls or in patients with autoimmune diseases such as systemic sclerosis or Siogren syndrome or systemic lupus erythematosus. In addition such anti-PsA peptide antibodies are detectable only in 3.3% of patients with psoriasis without PsA, and therefore they seem to be specific for PsA. Indeed, these antibodies are present in the vast majority (85%) of patients with PsA and are directed against peptide epitopes expressed in skin proteins (fibrilllin, desmocollin and keratin) and in N-RAP, highly expressed within the entheses. Increased knowledge about the extent of tendon and ligament involvement has led to the theory that enthesitis may be the primary event in PsA. Another interesting feature of the autoantibodies we have identified, is their ability to bind TLR2. Toll-like receptors (TLRs) play critical roles in the activation of innate immunity and we have previously shown that antibody triggering of TLR4 and TLR5 may contribute to autoimmunity [13], [28]. Recently it has been reported [29], [30] that T cell expression of TLR2 regulates Th17 cell responses and that stimulation with TLR2 agonists promotes Th17 differentiation and Th17 cytokine production. Based on these observation it is tempting to speculate that the anti-PsA peptide antibodies we describe here, may contribute to autoimmunity upon interaction with TLR2. In conclusion, we describe the identification of a novel antibody that is present in most of the patients with PsA. However, this antibody is also present in a few patients with rheumatoid arthritis and therefore it is not able to completely discriminate the two diseases. Nevertheless in the absence of other disease biomarkers our antibodies may represent an interesting tool for the diagnosis and follow up of patients with PsA.

## Supporting Information

S1 Figure **Western blot of HEK cells transfected with** ***TLR2*** **probed with mouse monoclonal antibodies.** Western blot analysis of HEK cells transfected with *TLR2* probed with an irrelevant mouse antibody (left hand side) and with the monoclonal antibody directed against TLR2 (right hand side).
(TIF)
