# Supplementary figures and images for: Crossreactive Autoantibodies Directed against Cutaneous and Joint Antigens Are Present in Psoriatic Arthritis
Source: PLoS One. 2014 Dec 16;9(12):e115424. doi: 10.1371/journal.pone.0115424 (PMC4267814; doi:10.1371/journal.pone.0115424)

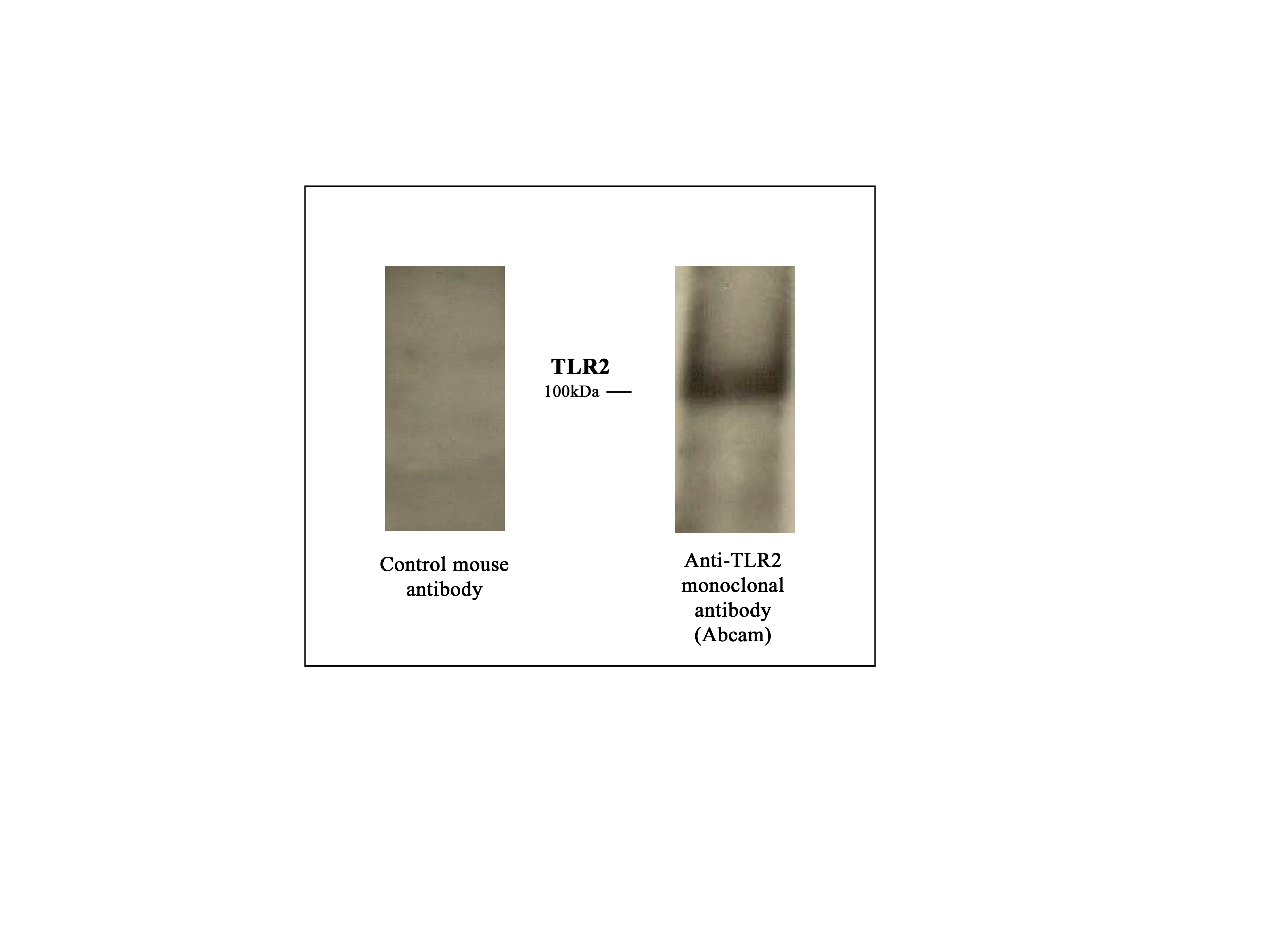

Supplement: S1 Figure — Western blot of HEK cells transfected with TLR2 probed with mouse monoclonal antibodies. Western blot analysis of HEK cells transfected with TLR2 probed with an irrelevant mouse antibody (left hand side) and with the monoclonal antibody directed against TLR2 (right hand side). (TIF) [file pone.0115424.s001.tif]
